# NERSkill.Id: Annotated dataset of Indonesian's skill entity recognition

**DOI:** 10.1016/j.dib.2024.110192

**Published:** 2024-02-14

**Authors:** Meilany Nonsi Tentua, Suprapto, Afiahayati

**Affiliations:** a Informatic, Sains and Technology Faculty, Universitas PGRI Yogyakarta, Indonesia; b Department of Computer Science and Electronics, Faculty of Mathematics and Natural Sciences, Universitas Gadjah Mada, Yogyakarta, Indonesia

**Keywords:** Natural language processing, Named entity recognition, Text mining, Skill entity recognition, Indonesian skill entity

## Abstract

NERSkill.Id is a manually annotated named entity recognition (NER) dataset focused on skill entities in the Indonesian language. The dataset comprises 418.868 tokens, each accompanied by corresponding tags following the BIO scheme. Notably, 15,51% of these tokens represent named entities, falling into three distinct categories: hard skill, soft skill, and technology. To construct this dataset, data were gathered from a job portal and subsequently processed using open-source libraries. Given the scarcity of annotated corpora for Indonesian, NERSkill.Id fills a significant void and offers immense value to multiple stakeholders. NLP researchers can harness the dataset's richness to advance skill entity recognition technology in the Indonesian language. Companies and recruiters can benefit by employing NERSkill.Id to enhance talent acquisition and job matching processes through accurate skill identification. Furthermore, educational institutions can leverage the dataset to adapt their courses and training programs to meet the evolving needs of the job market. This dataset can be effectively utilized for training and evaluating named entity recognition systems, empowering advancements in skill entity recognition for the Indonesian language.

Specifications Table

**Table:** 

Subject	Data science
Specific subject area	Skill Entity Recognition from job description in Indonesian Language
Data format	Raw Standardized
Type of data	Tabular
Data collection	The dataset was compiled using a combination of automated scraping, processing, and manual annotation techniques. Initially, job descriptions from various job vacancies listed on a job portal were extracted through the use of BeautifulSoup Python library. Subsequently, the gathered text files underwent manual annotation, where undergraduate of Informatics annotators labeled each token with the appropriate tag using a spreadsheet application. The final output was exported in a tabular txt format, following the BIO tagging scheme. Each row in the resulting dataset represents a token along with its corresponding tag, enabling the dataset to be effectively utilized for named entity recognition tasks.
Data source location	The Web
Data accessibility	Repository name: Mendeley Data Data identification number: 10.17632/5s8r9ndfvc.2 Direct URL to data: https://data.mendeley.com/datasets/5s8r9ndfvc/2

## 1 Value of the Data

• NERSkill.Id is the first annotated corpus for NER dataset focused on skill entities in the Indonesian language. It thus makes a valuable contribution to the available resources for Indonesian Language (NLP).
• This dataset is useful for computer NLP research community, companies, recruiters, and educational institutions
• This dataset can be used to evaluation or training in various tasks of skill recognition for transformer language models on the downstream task of NER.
• This dataset follows the BIO format and can thus be combined with other widely used corpora in standard to train large models.

## 2 Background

The primary objective of creating this dataset is to procure a precisely annotated Named Entity Recognition (NER) corpus specifically focused on skill entities in the Indonesian language. Although NERSkill.Id is relatively small in size, it has significant potential for fine-tuning language models. Additionally, it can be effectively combined with larger pre-existing corpora to facilitate the training of more comprehensive and adaptable mixed Indonesian models for various NLP tasks.

## 3 Data Description

**Fig. 1:**
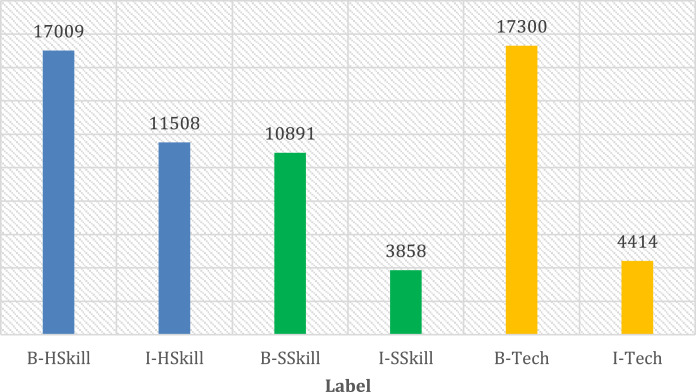
Distribution of annotation.

Following the processes of scraping, preprocessing, and annotation, the ultimate version of the dataset comprises 418.868 tokens. Notably, 15,51% of these tokens correspond to named entities. Before the annotation (tagging) stage, the sentences outlining job requirements undergo a tokenization process. The dataset categorizes named entities into three distinct classes: hard skill, soft skill, and technology [1]. Subsequently, these tokens are marked using the BIO format [2] (which stands for Beginning, Inside and Outside). The distribution of these specific named entities within the dataset is shown in Fig. 1.

Hard skill (HSkill) refers to specific abilities required for a job, typically listed under the qualifications section of a job vacancy [3]. Examples of hard skills include web design, computer programming, data analysis, and computer networking. Soft skill (SSkill) encompasses personality traits, personal attributes, and communication abilities needed to interact effectively with others and cultivate sensitivity towards the environment [3]. Examples of soft skills include teamwork, critical thinking, and conflict management. Technology (Tech) represents the type of methods used within Hard Skills [4]. Examples of technologies include C#, Python, MySQL, SQL Server, and Javascript. The annotation table is presented in ConLL2003 format, consisting of 2 columns: word and tag columns. The NERSkill-ID file is available in .txt format. Table 1 shows the description of colomns in NERSkill.Id. Table 2. illustrates the annotation format of the data performed by the NERSkill.ID dataset.

**Table 1:** Description of columns in NERSkill.Id dataset.

Column	Description
Word	A word, number, or punctuation mark representing one token
Tag	The tag assigned to the token according to the BIO tagging scheme

**Table 2:** Ilustration of annotation data.

Word	Tag
akrab	O
dengan	O
asp.net	B-Tech
core	I-Tech
(c#)	B-Tech
;	O
front-end	B-Hskill
frameworks	I-Hskill

## 3 Experimental Design, Materials and Methods

**Data scraping from job portal**. The data used to create the corpus were scraped from the Indeed^1^, Jobstreet^2^, loker.id^3^ dan Job.Id^4^. We used BeautifulSoup as Python library to extract data from indeed and Jobstreet. BeautifulSoup serves as a parser to separate HTML components into a sequence of easily readable elements. We collected manually for job description form loker.id and Job.id. From job portal, 4.394 job description were stored in text files. The full code of data scraping can be found on Mendeley Data^5^.

**Data annotation**. The text files obtained from the scraping phase were filtered by selecting data with a minimum of 5 words. We divided the files to be annotated into 4 sections. Each file will be annotated manually by 2 different annotators. Eight annotators, all undergraduate informatics students, were employed to annotate skills mentioned in job descriptions using a spreadsheet application. Before distributing the file, the involved annotators convened for a briefing session. The objective was to create a mutual comprehension of the designated tags, which encompassed hard skill, soft skill, and technology. Table 1 shows the annotation rules used for NERSkill.Id. Each sample was collectively deliberated upon, and the author assumed the role of the ultimate decision-maker. Following this, annotations were performed on the annotators' individual computers using a spreadsheet application. In cases of disagreement, the authors intervened to resolve any discrepancies and ensure data quality throughout the annotation process. Once the annotations were finalized, the output file was exported from the spreadsheet in txt format (Table 3).

**Table 3:** Annotation rules.

Entity Entity	Description
B-HSkill	Marks the beginning of a multi-word entity representing a Hard skill
I-HSkill	Refers to the following words within a Hard skill entity after B-HSkill
B-SSkill	Marks the beginning of a multi-word entity representing a Soft skill
I-SSkill	Refers to the following words within a Soft skill entity after B-SSkill
B-Tech	Marks the initiation of a multi-word entity representing a Technology
I-Tech	Refers to the words that follow within a Technology entity after B-Tech
O	Denotes words that do not belong to any recognized entity

**Reference results**. To test the usefulness of our data in training NER systems, we fine-tuning pretrained model language BERT [5], IndoBERT [6] and EBERT-RP [7] for NER modelling using NERSkill.Id. The model was trained on 5 epochs using a learning rate of 3e-5. The performance of the model on the test set, measured in terms of precision, recall, and F1-score is given in Table 4. We evaluate the model in token level and entity level.

**Table 4:** Evaluation of reference model on NERSkill.Id.

Tag	BERT [5]			IndoBERT [6]			EBERT-RP [7]		
	P	R	F1	P	R	F1	P	R	F1
B-HSkill	84%	89%	87%	83%	88%	85%	88%	92%	90%
B-SSkill	94%	96%	95%	93%	95%	94%	95%	98%	97%
B-Tech	91%	90%	91%	90%	92%	91%	94%	95%	94%
I-HSkill	85%	77%	81%	84%	79%	82%	89%	87%	88%
I-SSkill	90%	90%	90%	94%	91%	93%	93%	86%	90%
I-Tech	74%	69%	72%	77%	66%	71%	88%	76%	82%

*P= Precision; R=Recall; F1=F1-Score.

## Limitations

Not applicable.

## Ethics Statement

The data utilized to construct the dataset do not raise ethical issues, as they were sourced from a Job Portal rather than a social media platform or other sensitive data origins. Permission to employ data from the Job Portal was unnecessary. Our research did not involve any human or animal studies.

## CRediT authorship contribution statement

**Meilany Nonsi Tentua:** Methodology, Software, Investigation, Resources, Data curation, Writing – original draft, Visualization. **Suprapto:** Investigation, Validation, Writing – review & editing, Supervision. **Afiahayati:** Writing – review & editing, Investigation, Validation.

## Data Availability

NERSkill.Id (Original data) (Mendeley Data).

## Footnotes

1 https://id.indeed.com/.

2 https://www.jobstreet.co.id/.

3 https://www.loker.id/.

4 https://job.id/.

5 https://data.mendeley.com/datasets/5s8r9ndfvc/2.
